# Simple predictive scoring system for ventilator-associated pneumonia in trauma patients

**DOI:** 10.1186/cc9873

**Published:** 2011-03-11

**Authors:** N Saito, T Yagi, Y Hara, H Matsumoto, K Mashiko

**Affiliations:** 1 Chiba Hokusou Hospital, Nippon Medical School, Chiba, Japan

## Introduction

VAP is associated with high mortality in trauma patients. However, detailed data on the prediction of VAP in such patients are limited. We therefore conducted a retrospective study aimed at developing a VAP predictive scoring system for trauma patients.

## Methods

We retrospectively analyzed 187 consecutive patients with trauma who were ventilated for more than 72 hours between April 2006 and April 2010. VAP was diagnosed by CDC criteria. Patients were divided into the VAP group and non-VAP group and their clinical and laboratory data were compared by univariate analysis using the chi-square and Mann-Whitney U tests. Multivariate analysis using the stepwise method was used to identify predictors of VAP.

## Results

Victims of blunt trauma accounted for 90.9% of the sample population. The median age of the patients was 50 (32 to 67) years, the median injury severity score (ISS) was 29 (22 to 32), and the hospital mortality rate was 12.3%. Seventy patients were assigned to the VAP group (27.5/1,000 mechanical ventilator-days) and 117 to the non-VAP group. The independent predictors for VAP were thoracic cage trauma (odds ratio (OR) 2.5 (*P *= 0.02; 95% confidence interval (CI): 1.1 to 5.5)), history of chronic heart failure (CHF; OR 8.9 (*P *< 0.01; 95% CI: 2.4 to 33.0)), chronic obstructive pulmonary disease (COPD; OR 5.9 (*P *< 0.01; 95% CI: 1.9 to 18.3)), muscle relaxant (MR) use (OR 5.2 (*P *< 0.01; 95% CI: 1.7 to 15.3)), tracheal intubation (TI) in the prehospital setting (OR 4.7 (*P *< 0.01; 95% CI: 1.8 to 12.4)), use of a nasogastric (NG) tube (OR 6.5 (*P *< 0.01; 95% CI: 2.7 to 15.4)), cervical vertebrae external fixation (CVEF; OR 9.0 (*P *< 0.01; 95% CI: 2.2 to 36.7)), and ISS >25 (OR 5.0 (*P *< 0.01; 95% CI: 1.8 to 13.7)). Based on these results, we developed a VAP predictive scoring system. The following simplified clinical risk assessment tool was developed from the results of multivariate analysis, with scoring based on a cut-off point related to the adjusted odds ratio. VAP score: thoracic cage trauma = 2 points, CHF = 8 points, COPD = 5 points, MR use = 5 points, TI in the prehospital setting = 4 points, NG tube = 6 points, CVEF = 9 points, ISS >25 = 5 points. The area under the receiver operating characteristic curve for VAP in this scoring system was 0.847 (*P *< 0.001; 95% CI: 0.79 to 0.90). The cut-off value for this score according to the sensitivity specificity curve in relation to VAP was 15 points. Sensitivity was determined at 91.1%, and specificity 74.5%.

## Conclusions

The simple predictive scoring system developed for VAP in trauma will help physicians in the planning of early patient care.

